# Eugenic Legislation

**Published:** 1915-05

**Authors:** 


### Eugenic Legislation.

"Students of human heredity will view with satisfaction the appearance of Bulletin No. 82 of the University of Washington, which gives in 87 pages a summary of the laws of the several United States governing: I.—Marriage and divorce of the feeble-minded, the epileptic and the insane. II.—Asexualization. III.—Institutional commitment and discharge of the feeble-minded and the epileptic. After giving summaries of all the laws in question, in a clear and concise form, the authors terminate their work with four pages of temperate discussion of the entire subject of legislation intended to prevent the breeding of a degenerate race in the United States, pointing out that some of this legislation is so out of date, from a biological point of view, that it is worse than useless.

"'A half century ago,' they remark (page 82), 'educators still hoped by intensive education so to improve the mental condition of the feeble-minded as to make advisable their leaving the institution and assuming some, at least, of the duties of normal people. If this policy of the educators were to benefit society, it must have presupposed that acquired traits are, in the full meaning of the term, inherited. And this no one at that time doubted.

" 'Today we know that nearly all forms of mental deficiency are incurable, and most biologists believe that, in the full meaning of the term, acquired characters are not inherited. Legislatures, however, have other tasks than the study of modern biological theory, so that we see the opinions of Lamarck and of Seguin almost unchanged in many of the State laws.

" 'For instance, a number of institutions for the feeble-minded are intended only for those who "may be benefited by the instruction." The Illinois application blank indicates this. The Governor of New Jersey is empowered to remove any child who is not benefiting by the instruction given. Delaware, which sends its feeble-minded to the institutions of other States, orders its patients discharged when they may no longer receive benefit from training. In Kentucky the superintendent of the institution is supposed to return to the counties all patients, further attempts to educate whom will not prove beneficial to the State. Also, no child may be kept in the institution after arriving at such age and mental condition that he will be able to provide for himself.

" 'EDUCATION NOT A REMEDY.

" 'Regulations such as these are not often put to any greater use than necessary, but they still reflect the opinion of a few decades ago, that the superficially educated defective makes better material for parenthood than the uneducated defective. To discharge, unsterilized, the defective child, after having taught him habits of neatness and a few tricks that make his mental deficiency less noticeable, is worse than never to have put him in an institution. The same criticism applies, of course, to the special classes in the public schools.

" 'Among the marriage and divorce laws there is more Lamarckian biology. Kansas forbids the marriage (unless the wife is over 45 years of age) of children *born after a parent was insane.* Michigan and New Jersey demand the "cure" of defectives before marriage. Divorce is granted in Utah on the ground of insanity only when the condition is incurable.

" 'The Michigan asexualization law provides for sterilization when "there is no probability that the condition of such person so examined will improve to such an extent as to render procreation by such person advisable." A similar provision is found in the North Dakota law.'

"The authors go on to point out that some sterilization measures which are sound biologically may be quite wrong from a sociological point of view. In such a case, legislation must proceed very slowly. Finally they discuss briefly the whole question of whether laws to prevent bad breeding are feasible.

"'A frequent charge made against eugenic legislation is that it is unwise, that it is conceived in the isolation of the schools and will never bear the test of common use. Our attention is called to the many Utopias which have come to pass only in the minds of philosophers, and to the failure of most "ideal" communities due to their disregard of common sense premises.

"'Another objection to such laws is raised by the "all for love and the world well lost" school. In their opinion, even granted that by the exercise of the police power it were possible to realize this academic dream, the Eugenic State must necessarily be a cold-blooded breeding station where romance is the price paid for a better race.

"'Then there are theological objections. These apply especially to asexualization. It is the opinion of some people that although to operate on a man for his own good is justifiable, to operate on him for the good of society is to tamper with the plans of Providence. One also hears it said by people who are not avowedly actuated by theological considerations that such operations are "unnatural."

"'Lastly, the position is taken that eugenic laws are impracticable, that society will not tolerate them. To be sure, society has tolerated sex taboos and legal penalties much more onerous, but long standing has made them seem "natural."

"'OBJECTIONS EASILY ANSWERED.

"'The objections, then, to the legislative attempt to apply the known facts of biology to the betterment of human stock are (1) that the laws themselves would not accomplish what their authors predict, or (2) that the cost of reform is too great, or (3) that the laws are impious, or (4) that society is incorrigible and the laws cannot be enforced. In addition to these, Mr. G. B. Shaw has said that we do not know what type of human being we wish to develop, but as the opinion of society has in the past been a great factor in determining the survival of individuals, this objection holds only within certain limits. We are rather confident that there are certain characteristics we might wisely eliminate.

"'To take issue with these antagonistic criticisms of eugenic legislation is hardly the purpose of this publication, though a few of the more obvious rejoinders may be noted. The inheritance under certain conditions of mental deficiency is undisputed. Also the fact that mental defectives are undesirable seems evident. With such a backing for the project, the further social control of the defective deserves being tried out. Ultimately every social measure must survive or be eliminated according to the results of its practical application. To call legislation experimental is to praise it. If less legislation were regarded as final and more were measured by its results, we might have a better working code.

"'The second objection is due to ignorance of the history of sex taboo. When it is realized that the present limitation of our individual liberty in the matter of marriage is tacitly accepted by nearly everyone, and that certainly in the past much of this limitation was ridiculous, the objection to further limitation of a desirable sort falls to the ground, especially as in the change many of the present useless taboos will be eliminated. The taboo against incest, that of the Jews against all exogamy and our own against mating with certain alien races are not irksome to the average man. They rather add to the romance of marriage. An aristocratic pride of race is not antagonistic to a man's choice of a mate, but rather determines it and adds ultimately to his satisfaction. Certainly marriages are happiest when a wise taboo is respected.

"'It seems hardly necessary to meet the theological objections to progressive legislation along biological lines, as some theologians are among the ablest supporters of eugenic laws, and those who are not would not agree with the premises of eugenics. A bishop, recently addressing a university audience, condemned the eugenic movement, saying that the selection of the more fit only limited the field of religion, as it is the unfortunate who need religion most. Fortunately this is not a prevalent view in any church.

"'NEED FOR PUBLIC SENTIMENT.

"'In supposing that eugenic laws cannot be enforced, critics doubtless have in mind what they regard as analogous cases of laws which were enacted and became dead letters because the public did not feel their importance. Anti-tipping laws are examples of this, and the public certainly must demand such a law in advance of its enactment if it is not to die on their hands. Much eugenic legislation is, however, not prohibitory in character, but is permissive of certain powers on the part of the courts or of commissions appointed for a definite purpose. It is this sort of legislation that must necessarily work best, as it is not subject to violation and will continue to be used as long as even a few people are intelligently interested in it.

"'Whatever may be said against such laws as are presented in this pamphlet, and they are rightly subject to much criticism, it is evident that something must be done to diminish the number of mental defectives in our population. War among primitive people, poverty, disease, and capital punishment did a fairly thorough if not a very beautiful piece of work before we began to civilize them away. Some substitute has to be found for natural selection. Procreation of the undesirable must be prevented by means which are least cruel and least wasteful. These laws already in force in the several States indicate the growth of this opinion.'"—*The Journal of Heredity.* (See the proposal of Neo-Malthusianism in the March issue.)

